# Hyperandrogenism and Hyperestrogenism Secondary to Mixed Germ-Cell Testicular Tumor

**DOI:** 10.7759/cureus.27396

**Published:** 2022-07-28

**Authors:** Daniel I Sanford, Kian Asanad, Nima Nassiri, Jamal Nabhani

**Affiliations:** 1 Catherine and Joseph Aresty Department of Urology, Keck School of Medicine of University of Southern California, Los Angeles, USA

**Keywords:** germ-cell tumor, urology, hyperestrogenism, hyperandrogenism, testicular cancer

## Abstract

Testicular cancer with androgen and estrogen secretion is classically associated with Leydig cell tumors. Rare case reports have described this finding in germ-cell tumors along with signs of androgen and estrogen excess including gynecomastia and infertility. We report the case of a 19-year-old male with a non-seminomatous testicular germ-cell tumor found to have hyperandrogenism, hyperestrogenism, and suppression of central sex hormones. Similar findings may be underreported in the literature, and males with suspected testicular malignancy should be appropriately screened for signs of androgen and/or estrogen excess so they can be offered appropriate monitoring and counseling.

## Introduction

Testicular cancer is the most commonly diagnosed solid-organ malignancy in males between 15 and 44 years of age [1,2]. Over the last four decades, the incidence has been increasing; in 2021, there will be an estimated 9,470 new cases diagnosed with around 440 deaths in the United States alone [3,4].

Testicular tumors have also been found to secrete androgens and estrogens. This is classically associated with testicular stromal tumors, in particular, Leydig cell tumors. However, there have been rare reports of sex hormone secretion in germ-cell tumors [5,6]. These prior reports describe presenting symptoms of androgen and estrogen excess, including gynecomastia and infertility. Here, we present the case of a 19-year-old male with a non-seminomatous testicular germ-cell tumor found to have hyperandrogenism, hyperestrogenism, and suppression of central sex hormones.

## Case presentation

A 19-year-old male with no medical history presented to our clinic with three weeks of left testicular pain and swelling. He described a progressive increase in the size of his left testicle along with a pulling sensation in his left groin that radiated to the suprapubic region. He otherwise denied any recent weight changes, breast tenderness, fevers, chills, nausea, vomiting, or changes in bowel patterns. On physical examination, a firm baseball-sized left testicular mass was palpated. The mass was tender to palpation. The right testicle was within normal limits. The patient’s body mass index was 21.42 kg/m^2^. There was no evidence of gynecomastia on examination. Laboratory results revealed elevated serum tumor markers, as depicted in Table 1. Hormonal testing demonstrated significant elevations in testosterone (1,029 ng/dL) and estradiol (130 pg/dL) with undetectable levels of follicle-stimulating hormone (FSH) and luteinizing hormone (LH).

**Table 1:** Pre-surgery and three- and eight-week post-surgery serum tumor markers, androgen, and gonadotropin levels.

Test	Pre-surgery	Post-surgery three weeks	Post-surgery eight weeks	Reference value
Alpha-fetoprotein (ng/mL)	835	40.8	2.3	<8.3
Human chorionic gonadotropin (mlU/mL)	285	<1	<1	<5.0
Lactate dehydrogenase (U/L)	260	122	161	135–225
Testosterone (ng/dL)	1,029	408	n/a	249–836
Estradiol (pg/mL)	130	21	n/a	5–52
Follicle-stimulating hormone (mIU/mL)	<0.3	1.5	n/a	1.5–12.4
Luteinizing hormone (mIU/mL)	<0.3	5.4	n/a	1.7–8.6

**Figure 1:**
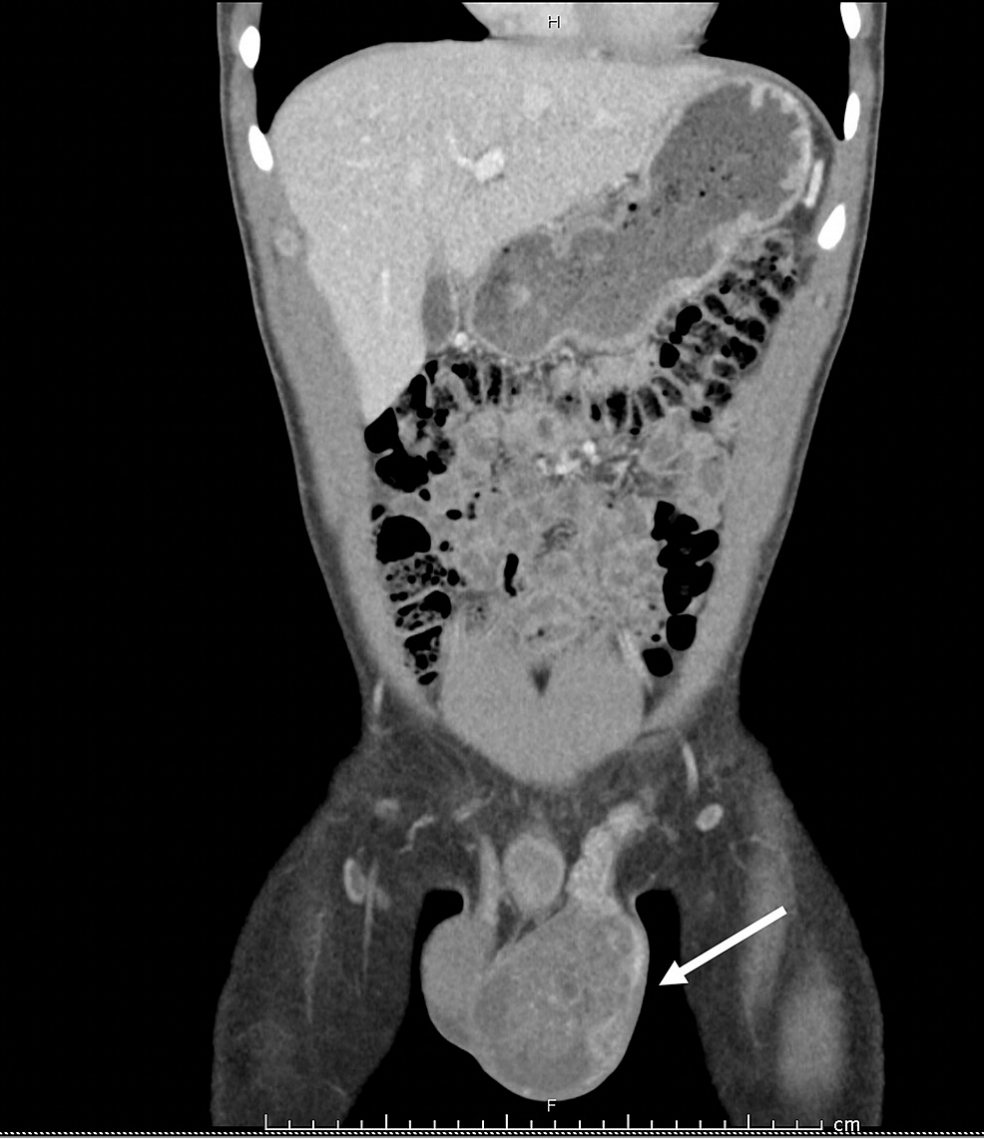
Computerized tomography scan of the abdomen and pelvis with contrast. Showing enlarged, heterogeneous left testicular mass measuring 7.0 × 6.0 cm.

**Figure 2:**
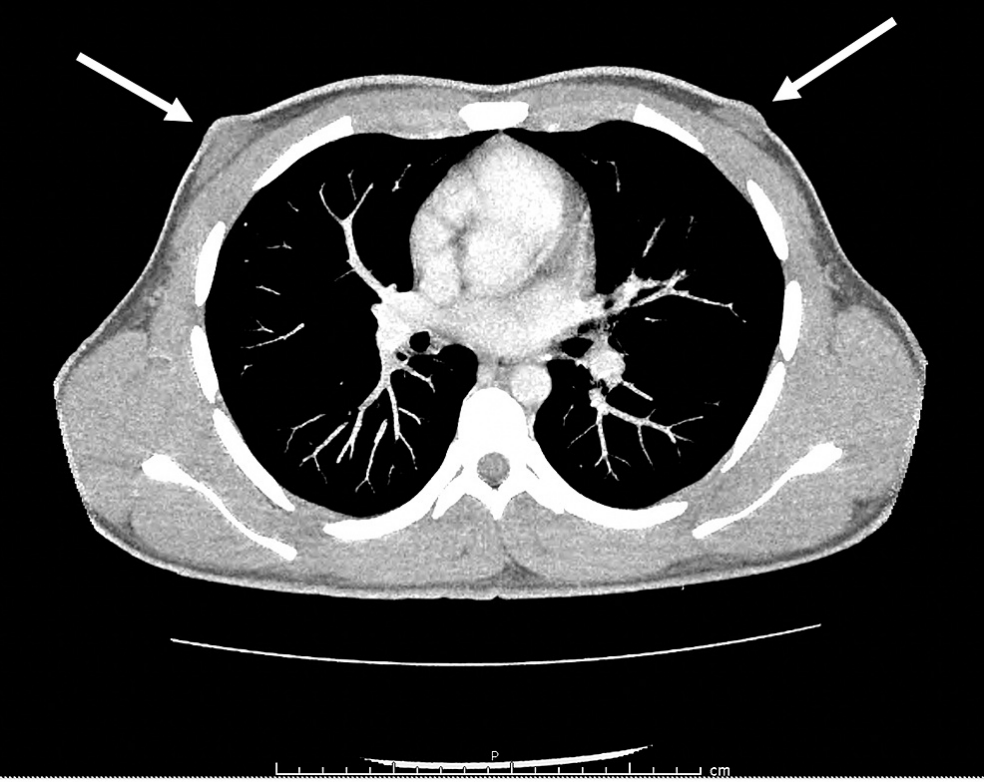
Computerized tomography scan of the thorax with contrast. Showing bilateral retro-areolar fatty lesions consistent with gynecomastia.

Scrotal ultrasonography demonstrated an irregularly shaped and enlarged left testicle with multiple cystic lucencies of varying size cysts. Computed tomography of the abdomen and pelvis displayed enlargement of the left testes to 7.0 × 6.0 cm but was otherwise without retroperitoneal lymphadenopathy or other metastatic diseases (Figure 1). Chest imaging was negative for pulmonary metastatic disease. Evidence of fat in the breast tissue was present suggestive of subclinical gynecomastia (Figure 2).

The patient underwent a standard radical left inguinal orchiectomy. The surgery was uncomplicated, and the patient had no postoperative complications. Final pathology demonstrated a mixed germ-cell tumor consisting of 50% immature teratoma, 35% seminoma, 10% yolk sac carcinoma, and 5% embryonal carcinoma. The pathologic tumor stage was pT1bNX with no lymphovascular invasion noted. The patient was followed closely after surgery. Follow-up tumor markers at three weeks and two months following surgery showed normalization of lab values (Table 1).

## Discussion

Testicular cancer is the most commonly diagnosed solid-organ malignancy in young males with an increasing incidence over the last four decades [1,2]. Initial workup for patients with suspected testicular cancer includes a detailed history and physical examination, testicular ultrasound, and serum tumor marker evaluation. Serum tumor markers are helpful in the workup and management of testicular cancer and have diagnostic importance, relevance for tumor staging, and an important role in monitoring for recurrence [7]. Serum androgen and estrogen levels can also provide insight when characterizing particular tumor types with elevated levels normally associated with Leydig cell tumors. However, there have been rare reports of androgen and estrogen secretion in germ-cell tumors [5,6].

Prior reports have documented symptoms of androgen excess in males found to have testicular germ-cell tumors. Presenting symptoms and physical examination findings included gynecomastia [6], infertility [8], precocious puberty [9], worsening acne, and increasing muscle mass [5]. It was suspected that elevated androgen levels were due to secondary Leydig cell hyperplasia from elevated human chorionic gonadotropin (hCG) [5]. Alternative explanations were a spontaneous synthesis of androgens by the tumor itself [6].

Prior studies have also documented estrogen-producing germ-cell tumors (testicular and mediastinal) leading to gynecomastia and male infertility [10]. Patients were noted to have a resolution of these symptoms following tumor resection. Similar to reports of androgen hypersecretion, hyperestrogenism was suspected to be due to hCG-induced Leydig cell hyperplasia with a paracrine mechanism of stimulation [6].

In this case, our patient with a testicular mass was found to have both androgen and estrogen excess. These elevated hormone levels were found following testing that we occasionally obtain prior to surgery to have a baseline because of results published by Djaladat et al. from our institution [6]. Prior to surgery, estrogen levels were nearly three times the normal limits while serum testosterone was elevated above the normal limits as well. We saw the suppression of central gonadotropins to undetectable levels. Our patient did not overtly present with symptoms of androgen or estrogen excess; however, a review of his computed tomography scan showed evidence of sub-areolar fat suggestive of subclinical gynecomastia. Following orchiectomy, he had normalization of his androgen and estrogen levels.

Our report adds to the current understanding of androgen and estrogen hormone elevations in testicular germ-cell tumors. No clear relationship between serum testosterone and estrogen levels exists in males with germ-cell tumors. Rieu et al. proposed that patients with hCG values between 5 and 3,500 IU/L had a positive correlation between hCG, estrogen, and testosterone levels [11]. Conversely, when hCG levels were greater than 3,500 IU/L, a negative correlation existed between hCG and serum testosterone [11]. In our patient, hCG levels were 285 IU/L pre-surgery, with elevated estrogen and testosterone, in agreement with the aforementioned trends.

What remains to be clarified is how common hyperandrogenism and hyperestrogenism are in germ-cell tumors as well as the precise mechanism by which they arise. Unless otherwise indicated, diagnostic algorithms do not characteristically call for testing for testosterone, estradiol, and gonadotropin levels when working up suspected testicular malignancy. The incidence of such findings may thus be underreported in the literature, particularly in patients presenting similar to ours with no overt symptoms of androgen/estrogen excess. Males with suspected testicular malignancy should be appropriately screened for signs of androgen and/or estrogen excess during the initial interview. Appropriate hormonal monitoring and counseling can thus be offered.

## Conclusions

There are limited reports of testicular germ-cell tumors with sex hormone hypersecretion in the literature. Here, we present the case of a non-seminomatous testicular germ-cell tumor found to have both androgen and estrogen excess leading to suppression of central gonadotropins and subclinical gynecomastia. Similar findings may be underreported in the literature as diagnostic algorithms do not recommend testing for testosterone, estradiol, and gonadotropin levels. Males with suspected testicular malignancy should be appropriately screened for signs of androgen and/or estrogen excess so they can be offered appropriate monitoring and counseling.
